# Long-term safety of dexamethasone sodium phosphate encapsulated in autologous erythrocytes in pediatric patients with ataxia telangiectasia

**DOI:** 10.3389/fneur.2024.1526914

**Published:** 2025-01-23

**Authors:** Mary Kay Koenig, Vincenzo Leuzzi, Riadh Gouider, Eppie M. Yiu, Barbara Pietrucha, Asbjørg Stray-Pedersen, Susan L. Perlman, Steve Wu, Trudy Burgers, Rupam Borgohain, Rukmini Mridula Kandadai, Isabelle Meyts, Giorgia Bucciol, Anaita Udwadia-Hegde, Ravi Yadav, Donna Roberts, Aaron Dane, Maureen Roden, Dirk Thye, Biljana Horn, Howard M. Lederman, William P. Whitehouse

**Affiliations:** ^1^Department of Pediatrics, Division of Child and Adolescent Neurology, University of Texas Health Science Center Houston McGovern Medical School, Houston, TX, United States; ^2^Department of Human Neuroscience, Unit of Child Neurology and Psychiatry, University of Rome La Sapienza, Rome, Italy; ^3^Department of Neurology, Clinical Investigation Centre Neurosciences and Mental Health, Razi University Hospital, Faculty of Medicine of Tunis, University of Tunis El Manar, Tunis, Tunisia; ^4^Faculty of Medicine of Tunis, University of Tunis El Manar, Tunis, Tunisia; ^5^Department of Neurology, Royal Children’s Hospital Melbourne, Neuroscience Research, Murdoch Children’s Research Institute, Melbourne, VIC, Australia; ^6^Neuroscience Research, Murdoch Children’s Research Institute, Melbourne, VIC, Australia; ^7^Department of Clinical Immunology, The Children’s Memorial Health Institute, Warsaw, Poland; ^8^Norwegian National Institute Unit for Newborn Screening, Division of Pediatric and Adolescent Medicine, Oslo University Hospitals, Oslo, Norway; ^9^Department of Neurology, University of California Los Angeles Medical Center UCLA, Los Angeles, CA, United States; ^10^Division of Neurology, Cincinnati Children’s Hospital Medical Center, Cincinnati, OH, United States; ^11^Department of Habilitation, Innlandet Hospital, Ottestad, Norway; ^12^Department of Neurology, Nizam’s Institute of Medical Sciences, Hyderabad, India; ^13^Parkinson’s Disease and Movement Disorder Research Centre, Citi Neuro Centre, Hyderabad, India; ^14^Department of Pediatrics, University Hospitals Leuven and Department of Microbiology Immunology and Transplantation, KU Leuven, Leuven, Belgium; ^15^Department of Neurology, Jaslok Hospital and Research Center, Mumbai, India; ^16^Department of Neurology, National Institute of Mental Health and Neurosciences, Bangalore, India; ^17^GEM Programming Solutions, Macclesfield, United Kingdom; ^18^Danestat Consulting Limited, Macclesfield, United Kingdom; ^19^Quince Therapeutics, South San Francisco, CA, United States; ^20^Division of Pediatric Allergy and Immunology, Johns Hopkins Hospital, Baltimore, MD, United States; ^21^School of Medicine, University of Nottingham, Nottingham, United Kingdom

**Keywords:** ataxia telangiectasia, dexamethasone sodium phosphate encapsulated in autologous erythrocytes, safety, adverse events, growth, bone mineral density

## Abstract

**Background:**

Dexamethasone sodium phosphate (DSP) encapsulated in autologous erythrocytes (EryDex) was developed as an alternative to standard glucocorticoids in an effort to eliminate chronic steroid toxicity while preserving efficacy. The primary objective of this report is to describe the safety of long-term use of EryDex in treatment of pediatric patients with ataxia telangiectasia.

**Methods:**

This is a post-hoc analysis of patients treated with EryDex for a minimum of 24 months in two prospective clinical trials. Outcomes include adverse events, growth, hemoglobin and serum iron, glucose levels, HbA1c, CD4+ lymphocytes, and bone mineral density.

**Results:**

Sixty-eight patients completed a minimum of 2 years of treatment with EryDex (mean treatment length 39 ± 11 months). Treatment-emergent adverse events (TEAE), reported in 67 (99%) out of 68 patients, were typically mild and did not cause discontinuation of treatment or death. Treatment-related TEAE were noted in 48 (71%) patients. Notable adverse events included transient pruritus reported in 23 (34%) patients and findings of low serum iron reported in 27 (40%) patients, while at baseline one fifth of patients had low serum iron. Anemia was reported in 9 (13%) patients. The mean hemoglobin level changed by −0.8 ± 1.0 g/dL after 6 months of therapy without subsequent decline. Longitudinal height and weight mean z-scores showed minimal change from baseline to month 24 for height (−0.06 ± 0.49), weight (−0.02 ± 0.71), and body mass index (0.03 ± 0.87). The mean bone mineral density (BMD) z-score showed a decline of 0.4 points over the 24 months of treatment. Values for glucose, HbA1c, cortisol, and CD4+ lymphocyte counts did not show clinically significant changes during prolonged treatment with EryDex.

**Conclusion:**

The most common treatment-related adverse events were transient infusion-related pruritus and iron deficiency. There was a decline in BMD which could not be distinguished from the natural course of disease. There were no adverse effects on height, weight and body mass index noted, as documented by stable z-scores throughout the 2 years of treatment. Adverse events typically observed with prolonged glucocorticoid use such as Cushingoid features, weight gain, hypertension, hirsutism, diabetes or stunted growth were rarely reported.

**Clinical trial registration:**

ClinicalTrials.gov, identifiers: NCT02770807 and NCT03563053.

## 1 Introduction

Ataxia telangiectasia is an autosomal recessive multisystem disorder caused by biallelic pathogenic variants in the *Ataxia Telangiectasia Mutated (ATM)* gene, located on chromosome 11q22·3 (1). The *ATM* gene codes for the protein of the same name (ATM), a member of the phosphoinositide 3-kinase-related kinases (PIKKs) which phosphorylates hundreds of proteins important in regulation of DNA repair, RNA-splicing, redox homeostasis, mitochondrial metabolic signaling, oxidative damage, and many other cellular functions (2). The most common clinical features of ataxia telangiectasia include neurological symptoms (ataxia with progressive cerebellar degeneration, involuntary movements, oculomotor apraxia, dysarthria), ocular and cutaneous telangiectasias, repeated infections contributing to chronic lung disease, delayed growth and pubertal development, and predisposition to cancers due to genetic instability and radiation sensitivity (3–5). According to the International Union of Immunological Societies classification of human inborn errors of immunity, ataxia telangiectasia is classified as a combined immunodeficiency of T and B lymphocytes with syndromic features (6). Lymphopenia is the most common immunologic abnormality, and it is seen in approximately 50% of patients. T cell functional defects comprise T cell activation and proliferation, abnormalities in T cell repertoire, and defects in cell signaling (7–11). IgA deficiency and IgG subclass deficiency and impaired antibody responses are seen in approximately 30–50% of patients (8, 9), while 10–20% of patients present with hyper IgM phenotype which is related to severe infections and shorter survival (12, 13). There are no approved therapies for this complex disease.

Glucocorticoids have been studied in ataxia telangiectasia since 2006 after it was observed that betamethasone ameliorated neurological symptoms when used in a patient with ataxia telangiectasia who had asthma. Neurological improvements were noticeable after 2 or 3 days of therapy, and after 4 weeks of treatment the improvement was dramatic: the disturbance of stance and gait was clearly reduced, and the control of the head and neck had increased, as had control of skilled movements. The neurological improvement was so great that the child was able to go up and down stairs (14). The efficacy of betamethasone was believed to be related to its antioxidative activity since it was shown that the patients with the best neurological response to therapy also had the highest intracellular glutathione levels (14, 15). Subsequent small studies of betamethasone showed efficacy but also suggested that adrenal suppression and reappearance of symptoms upon discontinuation of therapy limited glucocorticoid use (16–20).

Glucocorticoid effects are mediated by a ubiquitously expressed transcription factor, the glucocorticoid receptor, which influences the transcription rate of glucocorticoid-target genes, or interacts physically with other transcription factors regulating their transcriptional activity in a positive or negative fashion (21).

The therapeutic effect of dexamethasone in ataxia telangiectasia is likely mediated through various cellular mechanisms, many of which have been subject to recent investigation. Dexamethasone significantly elevates the levels of endogenous antioxidants, including glutathione and NADPH (14, 15). This elevation occurs via the NRF2-mediated upregulation of nuclear transcription for enzymes involved in antioxidant synthesis. Dexamethasone also directly inhibits KEAP1, integral to the KEAP1/NRF2 axis, at both transcriptional and post-translational levels (22). Additionally, dexamethasone facilitates the translation of short ATM variants that maintain residual kinase activity, while concurrently activating compensatory pathways that bolster ATM functions (23–27). These interactions substantiate multifaceted role of dexamethasone in cell regulation and protection against oxidative stress.

Although treatments with glucocorticoids can ameliorate a wide range of disorders, their use is limited by glucocorticoid resistance and toxicities which depend on their average and cumulative dose. Known side effects of glucocorticoids include behavioral or neurologic changes (emotional lability, insomnia, psychosis, pseudotumor cerebri); metabolic or gastrointestinal sequelae (weight gain, salt and water retention, hyperglycemia, diabetes, hypertension, fatty liver, pancreatitis, abdominal obesity, peptic ulcer, Cushingoid appearance); and endocrine effects (adrenal hypothalamic axis suppression affecting growth in height, sexual development and fertility). When glucocorticoids are tapered or omitted, fatigue, weakness, hypotension and mood change occur. Additional side effects include skin toxicities (hirsutism, acne, skin thinning and atrophy, bleeding and bruising, impaired would healing, hair thinning); effects on bones and muscles (myopathy, osteonecrosis, osteoporosis, impaired bone growth, fractures); increased risk of infections; and effects on the eyes (cataracts, glaucoma) (21).

Different modifications of synthetic glucocorticoids have been developed to optimize their therapeutic efficacy and improve the safety profile. One of the modifications is encapsulation of dexamethasone sodium phosphate (DSP) into autologous erythrocytes (EryDex), which is designed to provide continuous delivery of dexamethasone (28, 29). After an initial peak concentration, dexamethasone is released slowly from erythrocytes over a four-week period. The regimen is designed to preserve corticosteroid efficacy while avoiding side effects seen with daily steroid use (29). A phase two study of EryDex in children with ataxia telangiectasia suggested its efficacy (30, 31). Subsequently a large double-blind phase three clinical trial (ATTeST) evaluated efficacy of EryDex compared to placebo in 175 children with ataxia telangiectasia (32). The primary analysis of the initial 6 months of treatment in the ATTeST study showed no statistically significant effect of EryDex on neurological symptoms. The analysis of the per protocol population suggested treatment efficacy in patients who received treatment as outlined in the protocol. The mean difference in the modified International Cooperative Ataxia Rating Scale (mICARS) score was-2.21 (95% CI −4.05 to −0.37) points between patients receiving high dose EryDex and those receiving placebo in patients treated per protocol. Also, a subgroup analysis suggested a treatment effect of high-dose EryDex in 6–9-year-olds, with a mean difference in the mICARS of −2.79 (95% CI −5.09 to −0.48) points between treated younger patients and those receiving placebo. Following 6-months of treatment, there were no reports of Cushingoid appearance, hyperglycemia, hirsutism, hypertension, skin changes, growth delay, or behavioral changes related to steroid use in children treated with EryDex (32). However, the safety profile of long-term EryDex use has not been previously described.

One hundred and four patients from the ATTeST trial continued treatment on the open label extension trial (OLE). In this report, we include children receiving a minimum of 24 months of treatment, to allow for the longitudinal follow-up of growth. We describe reported adverse events and effects on growth, glucose metabolism, hemoglobin, serum iron, and bone health of prolonged use of DSP encapsulated in autologous erythrocytes in children with ataxia telangiectasia. Efficacy assessments of these patients are not included in this report.

## 2 Methods

### 2.1 Study design

This is a post-hoc analysis of patients with ataxia telangiectasia treated with EryDex in two clinical trials. The first trial was an international 22-center, randomized, double-blind, placebo-controlled, phase 3 clinical trial (ATTeST; NCT02770807, open from March 2017 until May 2021). The efficacy and safety results of the initial 6 months of therapy in this trial were previously published (32). After the initial 6-month period, participants were eligible to continue an additional 6-month, double-blind, placebo-controlled treatment, designed to collect longer-term safety and exploratory efficacy data. At the end of 1-year therapy in the ATTeST trial, patients desiring to continue therapy were offered high-dose EryDex in the open label extension trial (OLE; NCT03563053, open from June 2018 until September 2022), which continued in 17 ATTeST centers. The primary goal of the OLE trial was to evaluate long-term safety and tolerability of EryDex in this population. Both trials were conducted in accordance with the Good Clinical Practice guidelines of the International Conference on Harmonization (ICH) and the ethical principles of the Declaration of Helsinki. Written informed consent was obtained from all participants and/or parents or legal guardians for both trials. Both protocols were approved by regulatory authorities and ethics committees of all participating study sites.

### 2.2 Participants

The ATTeST trial included patients ≥6 years of age and > 15 kg followed at participating study centers, with clinical diagnosis of ataxia telangiectasia who had autonomous gait preserved (International Cooperative Ataxia Rating Scale [ICARS] walking score 0–4). Exclusion criteria included lymphopenia (CD4+ count <400/mm^3^ for 6-year-old participants, or < 150/mm^3^ for >6-year-olds); history of severe immunodeficiency; severe or unstable pulmonary, hepatic, or renal disease or uncontrolled diabetes. To continue treatment in the OLE study, participants must have completed treatment and the 12-month efficacy assessment in the double-blind ATTeST trial without serious or severe treatment-related adverse events and without any safety contraindications for study continuation.

To be included in this safety analysis, patients must have received a minimum of 24 months of active treatment with EryDex, either as treatment in the ATTeST and OLE studies, or in the OLE study only if they received placebo for the entire 12 months of the ATTeST trial. The 2-year minimum duration of treatment was required to allow for longitudinal assessment of growth in treated patients.

The EryDex System, manufactured by Quince Therapeutics (previously EryDel, S.p.A.), Medolla, Italy, is a point-of-care procedure which encapsulates DSP in autologous red blood cells, obtained by drawing 50 mL of whole blood from a patient. The process utilizes a series of hypotonic and hypertonic solutions to achieve diffusion and encapsulation of DSP within red blood cells (32). The procedure, performed at each treating institution using a dedicated automatic device system, takes ~1.5 h and it is repeated monthly during each treatment visit. The red blood cells with encapsulated DSP are reinfused into the patient immediately after obtaining a sample at the end of preparation to be used for sterility cultures. Subsequently, red blood cell phosphatases cleave dexamethasone from the prodrug resulting in a sustained level of active drug in patient’s circulation. During the ATTeST trial, participants were randomized between the low-dose EryDex (mean monthly dose of DSP encapsulated in red blood cells of 8.2 mg), high-dose EryDex (mean monthly DSP dose of 17.4 mg) or placebo, while all participants in the OLE trial received high-dose EryDex treatment (mean monthly DSP dose of 18.2 mg).

### 2.3 Study procedures

After the eligibility assessment and the initial 30-day screening period at the start of each trial, patients underwent baseline physical and neurological exams, and had safety laboratory tests, which were repeated monthly. Detailed neurological functioning was documented as an ICARS score at baseline and every 3 months in the ATTeST trial, and every 6 months in the OLE trial.

### 2.4 Outcomes

Safety data comprise TEAE, treatment-related TEAE, and serious adverse events (SAE). Adverse events of special interest (AESI) were widely defined in both trials and significantly overlapped with the list of TEAE, since the known adverse effects of glucocorticoids include almost all organ systems. Adverse events were coded using MedDRA version 24.0. Coding included system, organ class and preferred terms. Adverse events are reported for the entire treatment period from the first dose of EryDex until the end of treatment. Adverse events are presented as originally coded in the two trials. For AESI, when the same event was described in different categories, we presented the cumulative incidence of patients with an event, reported across different categories.

In addition to adverse events, the potential effect of EryDex on z-scores for height, weight, body mass index (BMI), and BMD of children with ataxia telangiectasia are described. BMD scans were obtained using dual energy x-ray absorptiometry (DXA) and included the spine and total body (less the head), following the guidelines provided in the 2013 International Society for Clinical Densitometry Official Pediatric Position (33). BMD scans were not obtained in all participating centers, due to the concern of potential effects of ionizing radiation on children with ataxia telangiectasia due to their known DNA repair defect.

We also summarize the results of safety laboratory tests used to assess steroid toxicities including cortisol, hemoglobin level, serum iron, random morning glucose, HbA1c, and absolute CD4 count. The last two measures were available only for the initial 12-month treatment period. All data available at baseline and within 2 months of each presented follow-up point (6, 12 and 24 months) were included. The ATTeST study used a central laboratory while the OLE safety tests were run in local laboratories. When standardization of laboratory values was not feasible (e.g., serum iron level), we present proportion of patients with reported abnormal values.

### 2.5 Statistical analysis

The adverse events are presented descriptively, summarizing the percentage of patients with an individual adverse event. Height, weight, and BMI measurements were converted to z-scores using LMS methods. That is z = (value/M^L-1)/(L*S) where value is the subject’s height, weight, or BMI, L is transformation for normality, M is median, and S is coefficient of variation. The LMS values are taken from the Centers for Disease Control and Prevention growth charts which are different for age (in months), and gender (34). These values are available for up to 20 years of age, thus data from patients older than 20 are not included in z-score presentations.

## 3 Results

Sixty-eight patients (median age 9 years at enrollment; quartile Q1 and Q3 7,11) completed ≥24 months of therapy with EryDex, and their characteristics are presented in Table 1. The mean duration of therapy was 39 ± 11 months. Twenty-nine (43%) out of 68 patients received a high-dose EryDex for the entire duration of their treatment, while the remainder had a combination of low-dose and high-dose EryDex. However, the duration of high-dose EryDex was longer than 18 months for 54 (79%) patients. The mean number of delivered EryDex treatments was 38.6 ± 14.48 per patient. The variability is the result of different lengths of treatment and not dose omission, since the mean number of omitted doses was 2.2 ± 1.4. Forty-six (68%) patients had at least one missed infusion.

**Table 1:** Characteristics of patients.

	Safety population
	*N* = 68
Age	
Mean (SD)	10.3 (5.36)
Median (Q1,Q3)	9 (7,11)
Age category	
6–9 years	40 (59%)
≥10 years	28 (41%)
Region	
India	5 (7%)
Europe, Australia, Tunisia	47 (69%)
United States	16 (24%)
Gender	
Male	38 (56%)
Female	30 (44%)
Genetic confirmation of A-T	
Genetically confirmed	65 (96%)
Not confirmed/missing	3 (4%)
Race	
White	59 (87%)
Asian	9 (13%)
Ethnicity	
Hispanic or Latino	4 (6%)
Non-Hispanic or Latino	64 (94%)
Randomization in ATTeST trial	
Low dose (LD) EryDex 12 months	32 (47%)
High dose (HD) EryDex 12 months	23 (34%)
Placebo 6 months, LD EryDex 6 months	4 (6%)
Placebo 6 months, HD EryDex 6 months	2 (3%)
Placebo 9 months, LD EryDex 3 months	3 (4%)
Placebo 9 month, HD EryDex 3 months	2 (3%)
Placebo 12 months	2 (3%)
Dose over the entire treatment period	
HD EryDex only	29 (43%)
LD and HD EryDex	39 (57%)
High Dose EryDex Duration	
>6–18 months	12 (18%)
>18 months	54 (79%)
Unknown	2 (3%)
Baseline Alpha-fetoprotein (ng/mL)	
Mean (SD)	268 (223)
Median (Q1, Q3)	194 (120,315)

HD, high dose; LD, low dose; Q, quartile; SD, standard deviation.

### 3.1 Treatment-emergent adverse events, treatment-related TEAE, and SAE

There were 1,426 reports of TEAE over the 39-month treatment period. Sixty-seven (99%) out of 68 patients had at least one TEAE reported, and 61 (90%) patients had an AESI reported. Forty-eight (71%) patients had treatment-related TEAE. Serious TEAE were reported in 17 (25%) patients, and in 1 (1%) patient serious TEAE was reported as treatment-related, as determined by the treating center’s principal investigator. One of the reported serious TEAE was seen in more than a single patient. There were no adverse events leading to discontinuation of treatment or death.

In Table 2, we describe TEAE which occurred in ≥10% of patients and their relationship to treatment. In Table 3, we list all SAE and their relationship to treatment. Supplementary Table S1 contains all TEAE reported in ≥2 patients, and their relationship to treatment. In the following text, we focus on more common TEAE by reported category, and we also present some selected less common TEAE because they describe issues with the investigational product and its infusion, or they are considered a typical steroid-related complication.

**Table 2:** Treatment-emergent adverse events (≥10% of patients) and treatment-related adverse events in safety population.

System Organ Class [n(%)] Preferred Term [n(%)] ≥2 patients by preferred term	TEAE (*N* = 68)	Treatment-related TEAE (*N* = 68)
Patients with Any TEAE [n (%)]*	67 (98.5)	48 (70.6)
Infections and Infestations	58 (85.3)	12 (17.6)
Upper Respiratory Tract Infection	24 (35.3)	2 (2.9)
Nasopharyngitis	23 (33.8)	2 (2.9)
Coronavirus Infection	16 (23.5)	1 (1.5)
Bronchitis	13 (19.1)	1 (1.5)
Gastroenteritis	7 (10.3)	0
Influenza	7 (10.3)	1 (1.5)
Rhinitis	7 (10.3)	0
General Disorders and Administration Site Conditions	40 (58.8)	12 (17.6)
Pyrexia	33 (48.5)	3 (4.4)
Fatigue	10 (14.7)	6 (8.8)
Investigations and Product Issues	51 (75.0)	17 (25.0)
Bacterial Test Positive	14 (20.6)	2 (2.9)
Product Contamination	11 (16.2)	0
Gastrointestinal Disorders	38 (55.9)	12 (17.6)
Vomiting	21 (30.9)	6 (8.8)
Diarrhea	19 (27.9)	2 (2.9)
Nausea	11 (16.2)	4 (5.9)
Respiratory, Thoracic, and Mediastinal Disorders	38 (55.9)	1 (1.5)
Cough	21 (30.9)	0
Rhinorrhea	13 (19.1)	0
Epistaxis	7 (10.3)	0
Oropharyngeal Pain	7 (10.3)	0
Injury, Poisoning, and Procedural Complications	37 (54.4)	22 (32.4)
Infusion Related Reaction	21 (30.9)	20 (29.4)
Fall	8 (11.8)	0
Skin and Subcutaneous Tissue Disorders	37 (54.4)	17 (25.0)
Pruritus	13 (19.1)	9 (13.2)
Rash	9 (13.2)	2 (2.9)
Erythema	8 (11.8)	3 (4.4)
Metabolism and Nutrition Disorders	28 (41.2)	22 (32.4)
Iron Deficiency	20 (29.4)	17 (25.0)
Nervous System Disorders	28 (41.2)	9 (13.2)
Headache	13 (19.1)	6 (8.8)
Neoplasms Benign, Malignant, and Unspecified (Including Cysts and Polyps)	8 (11.8)	1 (1.5)
Skin Papilloma	7 (10.3)	1 (1.5)

*Patients with multiple TEAEs are counted once.

**Table 3:** Serious treatment-emergent adverse events in safety population.

System Organ Class [n(%)] Preferred Term [n(%)] ≥2 patients by preferred term	Serious TEAE (*N* = 68)	Treatment-related SAE (*N* = 68)
Patients with Any Serious TEAE [n (%)]*	17 (25.0)	1 (1.5)
Investigations	9 (13.2)	1 (1.5)
Bacterial Test Positive	9 (13.2)	1 (1.5)
Infections and Infestations	2 (2.9)	
Lower Respiratory Tract Infection	1 (1.5)	
Viral Infection	1 (1.5)	
Nervous System Disorders	2 (2.9)	
Central Nervous System Lesion	1 (1.5)	
Dystonia	1 (1.5)	
Blood and Lymphatic Disorders	1 (1.5)	
Thrombocytopenic Purpura	1 (1.5)	
Gastrointestinal Disorders	1 (1.5)	
Dysphagia	1 (1.5)	
General Disorders and Administration Site Conditions	1 (1.5)	
Pyrexia	1 (1.5)	
Injury, Poisoning and Procedural Complications	1 (1.5)	
Forearm Fracture	1 (1.5)	
Neoplasms Benign, Malignant and Unspecified (Incl Cysts and Polyps)	1 (1.5)	
Odontogenic Cyst	1 (1.5)	
Respiratory, Thoracic and Mediastinal Disorders	1 (1.5)	
Pharyngeal Swelling	1 (1.5)	
Surgical and Medical Procedures	1 (1.5)	
Gastrointestinal Tube Insertion	1 (1.5)	
Vascular Disorders	1 (1.5)	
Aortic Stenosis	1 (1.5)	

### 3.2 Infections and respiratory, thoracic, and mediastinal disorders

Infections were the most common category of TEAE and were reported in 58 (85%) patients (Table 2). In 12 (18%) of patients an infection was considered treatment-related. The most common infections included upper respiratory tract infection [*N* = 24, 35%, treatment-related *N* = 2, 3%], nasopharyngitis [*N* = 23, 34%, treatment-related *N* = 2, 3%], coronavirus infection [*N* = 16, 24%, treatment-related *N* = 1, 1%], bronchitis [*N* = 13, 19%, treatment-related *N* = 1, 1%], gastroenteritis [*N* = 7, 10%, treatment-related *N* = 0], influenza [*N* = 7, 10%, treatment-related *N* = 1, 1%], and rhinitis [*N* = 7, 10%, treatment-related *N* = 0]. Less common infections, which are more frequently seen in patients on glucocorticoids, included pneumonia [*N* = 4, 6%, treatment-related *N* = 2, 3%], atypical pneumonia [*N* = 1, 1%, treatment-related *N* = 0], oral herpes [*N* = 4, 6%, treatment-related *N* = 1, 1%], herpes zoster [*N* = 3, 4%, treatment-related *N* = 2, 3%], and molluscum contagiosum and oral candidiasis [*N* = 2, 3%, for each respectively, treatment-related none]. There was also a report of a fungal skin infection, varicella zoster infection, herpes ophthalmic infection, herpes simplex infection, and vulvovaginal mycotic infection in one patient each, respectively. Only the fungal skin infection and the varicella zoster infections were considered treatment-related. There were no reports of sepsis, *Pneumocystis jirovecii*, or systemic fungal infections.

Thirty-eight (56%) patients had a TEAE reported in respiratory, thoracic and mediastinal disorder category, with only one (1%) being treatment-related. The most common adverse events included cough [*N* = 21, 31%, treatment-related *N* = 0], rhinorrhea [*N* = 13, 19%, treatment-related *N* = 0], epistaxis and oropharyngeal pain [*N* = 7 (10%), each respectively, treatment-related *N* = 0].

### 3.3 Non-infectious TEAE

In the general disorders category, the most common adverse events included pyrexia [*N* = 33, 49%, treatment-related *N* = 3, 4%] and fatigue [*N* = 10, 15%, treatment-related *N* = 6, 9%].

In the gastrointestinal disorders category the most common reports were vomiting [*N* = 21, 31%, treatment-related *N* = 6, 9%], diarrhea [*N* = 19, 28%, treatment-related *N* = 2, 3%], and nausea [*N* = 11, 16%, treatment-related *N* = 4, 6%].

In the skin and subcutaneous disorders category, pruritus not occurring during the drug infusion was reported in 4 (6%) patients. Other skin disorders included rash [*N* = 9, 13%, treatment-related *N* = 2, 3%], erythema [*N* = 8, 12%, treatment-related 3, 4%], alopecia [*N* = 4, 6%, treatment-related *N* = 1, 1%], acne [*N* = 3, 4%, treatment-related *N* = 0], and face swelling [*N* = 2, 3%, treatment-related *N* = 1, 1%].

In the musculoskeletal and connective tissue disorders category adverse events included osteopenia [*N* = 5, 7%, treatment-related *N* = 3, 4%] and osteoporosis [*N* = 5, 7%, treatment-related *N* = 2, 3%].

In the injury category, reported adverse events included falls [*N* = 8, 12%, treatment-related *N* = 0] and fractures. Fractures were reported in 5 (7%) patients (two hand fractures, two forearm fractures, one patient with both a wrist fracture and a foot fracture), and none were treatment-related.

Iron deficiency was reported in a total of 27 (40%) patients (among three categories including metabolism and nutrition, low serum iron, and low ferritin in laboratory investigations), and events were considered treatment-related in 17 patients (25%).

In the blood and lymphatic system disorder category, anemia was reported in 6 (9%) patients and iron deficiency anemia in 4 (6%) patients; however, one patient was reported under both categories, resulting in 9 (13%) patients with anemia. Seven out of 9 patients with anemia also had iron deficiency, reported as a separate AE. Anemia was considered treatment-related in 5 (7%) patients.

Headache was the common adverse event in the nervous system disorder category [*N* = 13, 19%, treatment-related *N* = 6, 9%]. In the psychiatric disorders category, reports included irritability [*N* = 5, 7%, treatment-related *N* = 2, 3%], depressed mood [*N* = 2, 3%, treatment-related *N* = 0], and emotional disorder [*N* = 2, 3%, treatment-related *N* = 2, 3%].

In the vascular category the main adverse event was flushing [*N* = 5, 7%, treatment-related *N* = 5, 7%].

Finally, skin papilloma was reported in 7 (10%) patients, treatment-related in 1 (1%). The distinction was not made between virally caused warts as opposed to skin tags.

### 3.4 Product-related adverse events and infusion-related adverse events

Positive sterility culture (listed as bacterial test positive and also listed under product contamination in Table 2) obtained at the end of processing, was reported in 23 (34%) patients and in 2 (3%) it was considered treatment-related. However, it did not result in symptoms of infection or positive blood cultures in patients who received those products. Root cause analysis showed that positive sterility cultures were related to inappropriate specimen handling, indeed bacteria identified were from the skin microbiome. After this finding and implementation of corrective action, positive sterility cultures attributed to incorrect sample handling were no longer reported as SAEs.

The most common infusion-related adverse event was genital or anal pruritus occurring during the infusion of EryDex, which was reported in a total of 23 (34%) patients across three categories (injury and procedural complications, skin and subcutaneous disorders, and reproductive). All cases were noted as treatment-related. Other infusion-related reactions included erythema of the cheeks for a day following infusion [*N* = 1, 1%, treatment-related], and vomiting which occurred during infusion [*N* = 1, 1%, not treatment-related].

Table 3 describes SAEs, which were reported in 17 (25%) patients and in 1 (1%) patient the SAE was considered treatment-related. Positive sterility test (listed as bacterial test positive) was the only SAE seen in more than one patient [*N* = 9, 13%].

### 3.5 Growth and BMD results

Steroid toxicity was also assessed by measuring longitudinal changes in patient z-scores for growth and BMD. Those results are presented in Table 4. Z-score for height, weight and BMI had minimal change over the 2-year treatment period (−0.06 ± 0.49 for height, −0.02 ± 0.71 for weight and 0.03 ± 0.87 for BMI).

**Table 4:** Change in height, weight, BMI, and bone mineral density after 24 months of treatment with EryDex.

	Baseline	24-months	Change from baseline
Height (cm) *N* = 66	134 (17.5)	142.9 (15.2)	8.9 (4.9)
Height (z-score) *N* = 66	−0.73 (1.38)	−0.79 (1.48)	−0.06 (0.49)
Weight (kg) *N* = 66	31.3 (15.2)	38.1 (17.0)	6.8 (5.0)
Weight (z-score) *N* = 66	−0.97 (1.85)	−0.99 (2.22)	−0.02 (0.71)
BMI (kg/m^2^) *N* = 66	16.46 (3.38)	17.8 (4.45)	1.34 (1.95)
BMI (z-score) *N* = 66	−0.75 (1.70)	−0.72 (1.94)	0.03 (0.87)
Bone mineral density (z-score) *N* = 37	−0.47 (1.21)	−0.87 (1.25)	−0.41 (0.95)

Data are mean (SD). BMI, body mass index.

Total body BMD declined by-0.41 ± 0.95 over the 2-year period. In 21 (57%) out of 37 patients who had BMD scan at baseline and month 24, the BMD z-score declined ≥0.5 over the 2-year follow-up. In 1 (3%) patient, the z-score improved ≥0.5, while in 15 (41%) the BMD z-score declined <0.5.

We also looked at the BMD values in five patients who sustained fractures during the observation period. In a patient with a hand fracture the BMD z-score of 0.7 was documented 2 months before the fracture, and the lowest BMD z-score of −0.4 was documented 11 months after the fracture. In the second patient with a hand fracture, the BMD z-score of −0.3 was documented 1 month before the fracture, and the lowest documented BMD z-score was −2.1, one year after the fracture. In a patient with a forearm fracture, the BMD z-score of −4 was documented 8 months before the fracture and was also the patient’s lowest BMD z-score. In the second patient with a forearm fracture, the BMD z-score was −0.9 1 month before the fracture, and that was also the lowest documented BMD z-score. One patient experienced both a foot fracture and a wrist fracture. The wrist fracture occurred 8 months prior to the foot fracture, and the BMD z-score of 0 was documented 4 months before the wrist fracture. The BMD z-score of −0.4 was documented 5 months before the foot fracture, and the lowest BMD score of −1 was documented 11 months after the foot fracture.

### 3.6 Hemoglobin, serum iron, glucose, cortisol, HbA1c and CD4+ count

At 6 months, mean hemoglobin level declined from 13.4 ± 1.4 g/dL to 12.6 ± 1.3 g/dL (*N* = 58) with a change from baseline of −0.8 ± 1.0 g/dL. At 24 months, mean hemoglobin level declined from the baseline of 13.3 ± 1.4 g/dL to 12.6 ± 1.1 g/dL (*N* = 62) with a 2-year change from baseline of-0.7 ± 1.0 g/dL.

At baseline, a low serum iron level was documented in 9/54 (17%) patients, while at 6-month follow-up serum iron was low in 24/54 (44%) patients. In 36 patients who had 2-year serum iron data, the baseline serum iron was low in 8/36 (22%) patients while at 24-month follow-up serum iron was low in 14/36 (39%) patients.

Data on random morning glucose levels, available for 53 patients at baseline and month 6, showed baseline mean glucose of 96.4 ± 16.52 mg/dL and the 6-month value of 94.42 ± 11.11 mg/dL, with a change of −1.95 ± 19.49 mg/dL. At 24 months, glucose levels were available for 41 patients. The baseline mean glucose level was 94.1 ± 10.75 mg/dL, and at 24 months it was 94.77 ± 19 mg/dL with a mean change from baseline of 0.64 ± 19.49 mg/dL.

First morning cortisol of ≤5 μg/dL was documented in three patients at baseline. In one of them, the subsequent cortisol was normal and in two patients, the subsequent levels were not done. Fifty-six *ad hoc* cortisol levels were drawn in 42 (62%) patients if adrenal suppression was clinically suspected or delay in treatment occurred. Low (<5 μg/dL) ad hoc cortisol level was documented in 3 (4%) patients. In only one instance the low cortisol was drawn as a first morning level. The low levels ranged between 3.9 and 4.6 μg/dL. Adrenocorticotropic hormone (ACTH) stimulation test was recommended if adrenal suppression was suspected. No patient in this study had an ACTH stimulation test.

HbA1c was available in 51 patients treated for 6 months. The baseline mean HbA1c was 5.1% ± 0.25, and at 6 months it was 5.2% ± 0.22 with a change from baseline 0.1% ± 0.20. Values for HbA1c were available for 22 patients at 12 months of treatment. The results showed baseline mean HbA1c of 5.2% ± 0.29 and 5.21% ± 0.22 at 12 months, resulting in a change from baseline of 0.02% ± 0.21.

CD4+ lymphocyte count was available for 48 patients at 6 months. The baseline mean absolute CD4+ count was 424 ± 263/μL, and at 6 months it was 410 ± 225/μL with a change of −14 ± 135. Similarly, in 24 patients with CD4+ results at 12 months, the baseline count was 400 ± 260/μL, and at 12 months it was 394 ± 219/μL with a change from baseline of −6 ± 107/μL.

## 4 Discussion

We evaluated the safety of DSP encapsulated in autologous erythrocytes in a large cohort of children with ataxia telangiectasia with a mean exposure to treatment of 39 months. We confirmed our previous 6-month observations that the safety profile of EryDex, is characterized by lack of typical steroid-related adverse events such as hypertension, hirsutism, or Cushingoid features (32).

A large number of TEAE were reported, which is expected in a population suffering from a progressive disease involving many systems and resulting in numerous events even in untreated patients. In the prospective ATTeST study that included a placebo arm, during the initial 6-month treatment period TEAE were reported in 73% of participants receiving low-dose of EryDex, 82% of participants receiving high-dose of EryDex and in 73% of participants receiving placebo (32). In this report covering a much longer treatment period, TEAE were reported in 99% of patients.

Patients with ataxia telangiectasia are known to have increased susceptibility to infections due to immunodeficiency, which causes low immunoglobulin levels and impaired antibody responses (35). Infections were the most common category of TEAE. However, serious infections were infrequent and systemic fungal infections were not seen. This is similar to what was previously observed in the ATTeST study, where infections were the most common category of TEAE (low-dose EryDex: 37%, high-dose EryDex: 30%, placebo: 22%), but serious infections were infrequent (32).

Our data from the ATTeST study indicated a similar distribution of TEAEs in EryDex-treated patients and those receiving placebo, with the exception of pruritus (32). Pruritus, which was described as a complication of infusion in approximately one third of long-term recipients of EryDex, is a known complication which occurs during intravenous infusion of dexamethasone. It is usually self-limiting and short in duration and can be ameliorated by reducing the infusion rate (36, 37). Another common TEAE was iron deficiency reported in 40% of patients in this study. Serum iron level was included in safety laboratory monitoring and low iron levels were subsequently reported as AE. Iron deficiency was likely the result of blood loss required for product preparation and safety monitoring, in particular in the double-blind part of the study that continued in lesser amount in the OLE. Regrettably, our studies did not fully evaluate iron status of children since ferritin levels were not available. More information on baseline serum iron and iron storage status in children with ataxia telangiectasia is required to fully understand observed changes in iron levels. It has been recently described that the ATM gene has an important role in iron metabolism, in particular in regulation of ferroptosis; however, clinical correlation of these findings in children with ataxia telangiectasia have not been yet described (38). Anemia or iron deficiency anemia was reported in 13% of patients, and although mean hemoglobin level changed by −0.8 g/dL by 6 months of therapy, there was no subsequent decline in hemoglobin level with prolonged therapy. Our finding of iron deficiency in 40% of treated patients warrants continuation of prospective surveillance for iron levels and iron stores in treated children. It is reassuring that SAEs were not common, and positive sterility culture after processing was the only SAE seen in more than one patient.

After 2 years of treatment with EryDex, height, weight, and BMI changed less than 0.1 z-scores from pre-treatment baseline. This is particularly notable because prepubertal children treated with chronic glucocorticoids experience decreases in height z-scores and increases in weight z-scores, with the effect on growth being proportional to duration and dose of glucocorticoids (39). Additionally, untreated children with ataxia telangiectasia show an annual decline in height of approximately 0.15 z-score, from age 1–16 years (40). Based on this information, a decline in height z-score of 0.3 points over 2 years may have been expected; however we observed a decline of 0.06. The weight gain expected with prolonged use of standard corticosteroids was reported as treatment-related in 3% of patients in this trial, while the weight z-score change in this trial was-0.02 over the 2 years. Very low frequency of adverse events related to growth or weight gain with longer-term use suggests that EryDex may be an attractive option for prepubertal children requiring chronic use of DSP.

We previously reported abnormal BMD z-scores in children with ataxia telangiectasia prior to initiation of EryDex, with a mean z-score of −1.08 ± 1.39 in 50 patients receiving low-dose EryDex, −0.98 ± 1.43 in 43 patients receiving high-dose treatment, and −1.18 ± 1.66 in 52 patients receiving placebo. After 6 months of therapy, the z-score declined in the high-dose group and placebo group and slightly increased in the low-dose EryDex group (32). After 2 years of treatment in this study, there was a decline in BMD of 0.41 z-score. We also documented 6 fractures in 5 patients, though none were considered treatment-related. However, only 2 out of 5 patients with fractures had BMD z-score of less than −1.5 documented at any time during their treatment. It is uncertain if the fractures were related to their bone health as opposed to difficulties with coordination related to the underlying disease, which would increase their risk of injury. Low BMD is related to the lack of ATM function. In mice with ataxia telangiectasia, it was postulated that ATM deficiency leads to osteoporosis, mainly as a result of hypogonadism-induced bone resorption together with compromised osteoblast differentiation (41). Another study described a group of children with rheumatoid arthritis who showed a decrease in ATM expression and a skewed B cell receptor repertoire, similar to the repertoire of patients with ataxia telangiectasia (42). Altered ATM function in B cells was associated with decreased osteoprotegerin and increased RANKL production. These changes favor bone loss and correlate with a higher prevalence of erosive disease in patients with rheumatoid arthritis who show impaired ATM function (42). While children with ataxia telangiectasia treated with EryDex grew more than expected based on natural course of the disease, EryDex treatment did not change the loss of BMD expected with this disease.

Safety measures, such as glucose, HbA1c, and CD4+ lymphocyte count did not show clinically significant changes, contrary to what would be expected with prolonged treatment with standard glucocorticoids. Very few patients had abnormal cortisol levels, and the investigators did not feel a need to further investigate adrenal insufficiency.

## 5 Conclusion

In this report on the long-term use and safety of EryDex, side effects typically attributed to chronic glucocorticoid use, such as hypertension or hirsutism, were not observed. Treatment-related Cushingoid face (face swelling) was reported in 1 (1%) patient while treatment-related weight gain was reported in 2 (3%) patients. Pruritus during the infusion of the product and low iron levels were the most common reported adverse events. There were no deaths or discontinuations of treatment related to AEs. There were no identified adverse effects on glucose metabolism, despite prolonged exposure to EryDex. Changes in BMD require further studies and comparisons with untreated ataxia telangiectasia patients. Preserved height and weight z-scores indicate a lack of stunted growth or weight gain typically seen with prolonged glucocorticoid use in children. The safety profile described herein suggests that EryDex may be a promising agent for use in pediatric patients with a disease that requires prolonged exposure to glucocorticoids.

## Data Availability

The raw data supporting the conclusions of this article will be made available by the authors, without undue reservation.
