# Supplementary material for: Long-term safety of dexamethasone sodium phosphate encapsulated in autologous erythrocytes in pediatric patients with ataxia telangiectasia
Source: Front Neurol. 2025 Jan 23;15:1526914. doi: 10.3389/fneur.2024.1526914 (PMC11800360; doi:10.3389/fneur.2024.1526914)
Supplement: Supplementary file 1 [file Table_1.DOCX]

Supplementary Material

# Table S1. Treatment-emergent adverse events (≥ 2 patients by MedDRA preferred term), treatment-related adverse events, and serious treatment-emergent adverse events in safety population

| **System Organ Class [n(%)]**^b^ **Preferred Term [n(%)]**  ≥2 patients by preferred term | **TEAE (N=68)** | **Treatment-related TEAE (N=68)** | **Serious TEAE**  **(N=68)** |
| --- | --- | --- | --- |
| Patients with Any TEAE [n (%)]* | 67 (98.5) | 48 (70.6) | 17 (25) |
| Infections and Infestations | 58 (85.3) | 12 (17.6) | 2 (2.9) |
| Upper Respiratory Tract Infection | 24 (35.3) | 2 (2.9) |  |
| Nasopharyngitis | 23 (33.8) | 2 (2.9) |  |
| Coronavirus Infection | 16 (23.5) | 1 (1.5) |  |
| Bronchitis | 13 (19.1) | 1 (1.5) |  |
| Gastroenteritis | 7 (10.3) | 0 |  |
| Influenza | 7 (10.3) | 1 (1.5) |  |
| Rhinitis | 7 (10.3) | 0 |  |
| Otitis Media | 5 (7.4) | 1 (1.5) |  |
| Urinary Tract Infection | 5 (7.4) | 1 (1.5) |  |
| Conjunctivitis | 4 (5.9) | 1 (1.5) |  |
| Ear Infection | 4 (5.9) | 0 |  |
| Oral Herpes | 4 (5.9) | 1 (1.5) |  |
| Pharyngitis Streptococcal | 4 (5.9) | 0 |  |
| Pneumonia | 4 (5.9) | 2 (2.9) |  |
| Respiratory Tract Infection | 4 (5.9) | 1 (1.5) |  |
| Bronchitis Bacterial | 3 (4.4) | 0 |  |
| Herpes Zoster | 3 (4.4) | 2 (2.9) |  |
| Hordeolum | 3 (4.4) | 0 |  |
| Sinusitis | 3 (4.4) | 0 |  |
| Eye Infection | 2 (2.9) | 0 |  |
| Folliculitis | 2 (2.9) | 1 (1.5) |  |
| Lower Respiratory Infection | 2 (2.9) | 0 | 1 (1.5) |
| Molluscum Contagiosum | 2 (2.9) | 0 |  |
| Oral Candidiasis | 2 (2.9) | 0 |  |
| Pharyngitis | 2 (2.9) | 0 |  |
| Skin Infection | 2 (2.9) | 0 |  |
| Tonsillitis | 2 (2.9) | 0 |  |
| Tooth infection | 2 (2.9) | 0 |  |
| Viral Infection | 2 (2.9) | 0 | 1 (1.5) |
| General Disorders and Administration Site Conditions | 40 (58.8) | 12 (17.6) | 1 (1.5) |
| Pyrexia | 33 (48.5) | 3 (4.4) | 1 (1.5) |
| Fatigue | 10 (14.7) | 6 (8.8) |  |
| Feeling Hot | 2 (2.9) | 0 |  |
| Pain | 2 (2.9) | 1 (1.5) |  |
| Investigations | 40 (58.8) | 17 (25.0) | 9 (13.2) |
| Bacterial Test Positive^b^ | 14 (20.6) | 2 (2.9) | 9 (13.2) |
| Blood Iron Decreased^a^ | 6 (8.8) | 1 (1.5) |  |
| Blood Lactate Dehydrogenase  Increased | 5 (7.4) | 4 (5.9) |  |
| Coronavirus Test Positive | 5 (7.4) | 0 |  |
| Bone Density Decreased | 4 (5.9) | 2 (2.9) |  |
| Serum Ferritin Decreased^a^ | 4 (5.9) | 1 (1.5) |  |
| Body Temperature | 3 (4.4) | 0 |  |
| CD4 Lymphocytes Decreased | 3 (4.4) | 1 (1.5) |  |
| White Blood Cell Count Decreased | 3 (4.4) | 2 (2.9) |  |
| Blood Bilirubin Increased | 2 (2.9) | 1 (1.5) |  |
| Blood Triglycerides Increased | 2 (2.9) | 1 (1.5) |  |
| Breath Sounds Abnormal | 2 (2.9) | 0 |  |
| Low Density Lipoprotein Increased | 2 (2.9) | 0 |  |
| Neutrophil Count Decreased | 2 (2.9) | 2 (2.9) |  |
| Protein Urine Present | 2 (2.9) | 1 (1.5) |  |
| Vitamin D Decreased | 2 (2.9) | 0 |  |
| Weight Increased | 2 (2.9) | 2 (2.9) |  |
| Gastrointestinal Disorders | 38 (55.9) | 12 (17.6) | 1 (1.5) |
| Vomiting | 21 (30.9) | 6 (8.8) |  |
| Diarrhea | 19 (27.9) | 2 (2.9) |  |
| Nausea | 11 (16.2) | 4 (5.9) |  |
| Abdominal Pain | 5 (7.4) | 1 (1.5) |  |
| Aphthous Ulcer | 3 (4.4) | 1 (1.5) |  |
| Constipation | 2 (2.9) | 0 |  |
| Dyspepsia | 2 (2.9) | 1 (1.5) |  |
| Gastrointestinal Disorder | 2 (2.9) | 1 (1.5) |  |
| Toothache | 2 (2.9) | 0 |  |
| Respiratory, Thoracic, and Mediastinal Disorders | 38 (55.9) | 1 (1.5) | 1 (1.5) |
| Cough | 21 (30.9) | 0 |  |
| Rhinorrhea | 13 (19.1) | 0 |  |
| Epistaxis | 7 (10.3) | 0 |  |
| Oropharyngeal Pain | 7 (10.3) | 0 |  |
| Nasal Congestion | 5 (7.4) | 0 |  |
| Productive Cough | 4 (5.9) | 0 |  |
| Catarrh | 2 (2.9) | 0 |  |
| Nasal Obstruction | 2 (2.9) | 0 |  |
| Sneezing | 2 (2.9) | 0 |  |
| Injury, Poisoning, and Procedural Complications | 37 (54.4) | 22 (32.4) | 1 (1.5) |
| Infusion Related Reaction^c^ | 21 (30.9) | 20** (29.4) |  |
| Fall | 8 (11.8) | 0 |  |
| Contusion | 5 (7.4) | 1 (1.5) |  |
| Ligament Sprain | 4 (5.9) | 0 |  |
| Skin Abrasion | 3 (4.4) | 0 |  |
| Face Injury | 2 (2.9) | 0 |  |
| Forearm Fracture | 2 (2.9) | 0 | 1 (1.5) |
| Hand Fracture | 2 (2.9) | 0 |  |
| Procedural Pain | 2 (2.9) | 0 |  |
| Skin Laceration | 2 (2.9) | 0 |  |
| Skin and Subcutaneous Tissue Disorders | 37 (54.4) | 17 (25.0) |  |
| Pruritus^c^ | 13 (19.1) | 9 (13.2) |  |
| Rash | 9 (13.2) | 2 (2.9) |  |
| Erythema | 8 (11.8) | 3 (4.4) |  |
| Skin Lesion | 5 (7.4) | 3 (4.4) |  |
| Alopecia | 4 (5.9) | 1 (1.5) |  |
| Acne | 3 (4.4) | 0 |  |
| Dermatitis | 3 (4.4) | 0 |  |
| Dry Skin | 3 (4.4) | 0 |  |
| Eczema | 3 (4.4) | 0 |  |
| Hyperhidrosis | 3 (4.4) | 1 (1.5) |  |
| Perioral Dermatitis | 2 (2.9) | 0 |  |
| Rash Erythematous | 2 (2.9) | 0 |  |
| Skin Depigmentation | 2 (2.9) | 0 |  |
| Swelling Face | 2 (2.9) | 1 (1.5) |  |
| Metabolism and Nutrition Disorders | 28 (41.2) | 22 (32.4) |  |
| Iron Deficiency^a^ | 20 (29.4) | 17 (25.0) |  |
| Increased Appetite | 4 (5.9) | 4 (5.9) |  |
| Vitamin D Deficiency | 4 (5.9) | 0 |  |
| Decreased Appetite | 3 (4.4) | 0 |  |
| Hypocalcemia | 2 (2.9) | 2 (2.9) |  |
| Nervous System Disorders | 28 (41.2) | 9 (13.2) | 2 (2.9) |
| Headache | 13 (19.1) | 6 (8.8) |  |
| Dizziness | 6 (8.8) | 2 (2.9) |  |
| Ataxia | 4 (5.9) | 0 |  |
| Balance Disorder | 2 (2.9) | 0 |  |
| Drooling | 2 (2.9) | 0 |  |
| Paraesthesia | 2 (2.9) | 2 (2.9) |  |
| Syncope | 2 (2.9) | 0 |  |
| Musculoskeletal and Connective Tissue Disorders | 25 (36.8) | 3 (4.4) |  |
| Arthralgia | 6 (8.8) | 0 |  |
| Osteopenia | 5 (7.4) | 3 (4.4) |  |
| Osteoporosis | 5 (7.4) | 2 (2.9) |  |
| Foot Deformity | 4 (5.9) | 0 |  |
| Pain in Extremity | 4 (5.9) | 0 |  |
| Scoliosis | 4 (5.9) | 0 |  |
| Joint Swelling | 2 (2.9) | 0 |  |
| Muscle Spasms | 2 (2.9) | 0 |  |
| Toe Walking | 2 (2.9) | 0 |  |
| Blood and Lymphatic System Disorders | 14 (20.6) | 9 (13.2) | 1 (1.5) |
| Anemia^d^ | 6 (8.8) | 2 (2.9) |  |
| Iron Deficiency Anemia^d^ | 4 (5.9) | 3 (4.4) |  |
| Neutropenia | 3 (4.4) | 3 (4.4) |  |
| Leukocytosis | 2 (2.9) | 1 (1.5) |  |
| Leukopenia | 2 (2.9) | 1 (1.5) |  |
| Eye Disorders | 14 (20.6) | 2 (2.9) |  |
| Conjunctivitis | 3 (4.4) | 0 |  |
| Chalazion | 2 (2.9) | 0 |  |
| Psychiatric Disorders | 12 (17.6) | 5 (7.4) |  |
| Irritability | 5 (7.4) | 2 (2.9) |  |
| Depressed Mood | 2 (2.9) | 0 |  |
| Emotional Disorder | 2 (2.9) | 2 (2.9) |  |
| Enuresis | 2 (2.9) | 0 |  |
| Vascular Disorders | 12 (17.6) | 7 (10.3) | 1 (1.5) |
| Flushing | 5 (7.4) | 5 (7.4) |  |
| Hematoma | 2 (2.9) | 0 |  |
| Hypotension | 2 (2.9) | 1 (1.5) |  |
| Product Issues | 11 (16.2) | 0 |  |
| Product Contamination^b^ | 11 (16.2) | 0 |  |
| Neoplasms Benign, Malignant, and Unspecified (Including Cysts and Polyps) | 8 (11.8) | 1 (1.5) | 1 (1.5) |
| Skin Papilloma | 7 (10.3) | 1 (1.5) |  |
| Ear and Labyrinth Disorders | 7 (10.3) | 1 (1.5) |  |
| Otorrhea | 2 (2.9) | 0 |  |
| Surgical and Medical Procedures | 5 (7.4) |  | 1 (1.5) |
| Congenital, Familial, and Genetic Disorders | 4 (5.9) | 0 |  |
| Muscular Dystrophy | 2 (2.9) | 0 |  |
| Reproductive System and Breast Disorders | 3 (4.4) | 3 (4.4) |  |
| Pruritis Genital | 2 (2.9) | 2 (2.9) |  |

Abbreviations: MedDRA= Medical Dictionary for Regulatory Activities; TEAE=treatment emergent adverse events.

- *Patients with multiple TEAEs are counted once

^a^  27 individual patients with low iron values, reported among terms: low ferritin, blood iron decreased (investigations) and iron deficiency (metabolism and nutrition)

^b^ 23 individual patients had positive sterility culture reported in 2 categories: investigations “bacterial test positive” and product issues “product contamination”.

^c^  Total of 23 patients had pruritus (generalized/anal/genital) during infusion, that was reported as treatment-related. **one patient had an episode of vomiting during the infusion of product which was reported as unrelated to the infusion

^d^ One patient was reported under anemia and iron deficiency anemia
